# Endogenous Gene Regulation as a Predicted Main Function of Type I-E CRISPR/Cas System in *E. coli*

**DOI:** 10.3390/molecules24040784

**Published:** 2019-02-21

**Authors:** Bojan Bozic, Jelena Repac, Marko Djordjevic

**Affiliations:** Institute of Physiology and Biochemistry, Faculty of Biology, University of Belgrade, 11000 Belgrade, Serbia; bbozic@bio.bg.ac.rs (B.B.); jelenag@bio.bg.ac.rs (J.R.)

**Keywords:** CRISPR/Cas, non-canonical CRISPR functions, CRISPR adaptation, transcription regulation, bioinformatics

## Abstract

CRISPR/Cas is an adaptive bacterial immune system, whose CRISPR array can actively change in response to viral infections. However, Type I-E CRISPR/Cas in *E. coli* (an established model system), appears not to exhibit such active adaptation, which suggests that it might have functions other than immune response. Through computational analysis, we address the involvement of the system in non-canonical functions. To assess targets of CRISPR spacers, we align them against both *E. coli* genome and an exhaustive (~230) set of *E. coli* viruses. We systematically investigate the obtained alignments, such as hit distribution with respect to genome annotation, propensity to target mRNA, the target functional enrichment, conservation of CRISPR spacers and putative targets in related bacterial genomes. We find that CRISPR spacers have a statistically highly significant tendency to target (i) host compared to phage genomes, (ii) one of the two DNA strands, (iii) genomic dsDNA rather than mRNA, (iv) transcriptionally active regions, and (v) sequences (*cis*-regulatory elements) with slower turn-over rate compared to CRISPR spacers (*trans*-factors). The results suggest that the Type I-E CRISPR/Cas system has a major role in transcription regulation of endogenous genes, with a potential to rapidly rewire these regulatory interactions, with targets being selected through naïve adaptation.

## 1. Introduction

Bacteria use repetitive genomic elements termed CRISPR (clustered regularly interspaced short palindromic repeats), together with CRISPR associated (Cas) proteins to target and destroy invading DNA. In terms of applications, CRISPR/Cas has revolutionized biotechnology, mainly as a versatile genome engineering tool [1]. These biotechnology applications are based on an understanding of native CRISPR/Cas systems (in bacterial/archaeal cells), where future biotechnology applications also depend on the further understanding of native roles of CRISPR/Cas systems [2]. For example, editing of the genome sequence is irreversible, and may be controversial in some applications [3]. On the other hand, the system can also be used to regulate gene expression [4,5,6], which has a potential to transiently or stably control gene expression levels without changing the gene sequence itself (see e.g., [7] and references therein). In such applications, at least some safety and ethical concerns (such as irreversible off-target effects) related to CRISPR-based genome editing approaches are absent [4]. Consequently, it may be of significant interest to understand CRISPR/Cas functions in bacterial cells other than its canonical immune function, in particular, the functions related to the regulation of endogenous gene expression.

On the other hand, it is widely considered that the main function of CRISPR/Cas is to protect prokaryotic cells from exogenous genetic material, such as phage or plasmid DNA [8,9]. CRISPR array is composed of alternating repeat and unique spacer sequences. New spacers, which correspond to invader DNA fragments [9,10,11], are added onto the leading (upstream) segment of the array during the process of adaptation. The spacers, which are expressed as crRNAs, therefore, represents the memory of encountered infective agents [12]. Upon a re-occurring infection, the foreign DNA is specifically recognized by crRNAs, which are derived from spacer sequences, and eventually cleaved by associated Cas (CRISPR-associated) proteins, which together constitute interfering RnpC (ribonucleoprotein complex) complex. Such a defense function of CRISPR/Cas is often called canonical.

However, new evidence also indicates important non-canonical CRISPR/Cas functions. Such non-canonical functions have first been assigned to Type II systems and connected to either Cas proteins (Cas2 in *Legionella pneumophila*, Cas9 in *Campylobacter jejuni* [13,14]), or novel CRISPR/Cas-associated small RNAs (scaRNA [15]), which are functional elements unrelated to spacer content, per se. However, a recently discovered non-canonical activity of the Type I-F system from *Pseudomonas aeruginosa*, directed against an endogenous mRNA, was shown to be crRNA-mediated—a likely consequence of the aforementioned spacer acquisition processes, that operate on the chromosome [16]. Our recent findings also indicate that small RNAs associated with non-canonical functions appear widespread in Type II systems [17]. Overall, these examples suggest a wider spectrum of CRISPR/Cas targets, and the existence of alternative routes for spacer acquisition. These alternative routes may be provided by naïve adaptation, which was shown to preferentially sample spacers from chromosome over extrachromosomal elements (likely due to a much larger chromosome size) [18]. This acquisition usually happens on the actively replicated/transcribed genomic regions [19,20]. In a recent work, the authors and other collaborators, also showed that sequence determinants for naïve adaptation are elusive, and likely correspond to some of the more global sequence properties, such as generation of adaptation substrates through DNA transcription or replication [21]. In the literature, somewhat different trends are also reported for naïve adaptation [10,22], in particular, that the spacers are preferentially sampled from foreign vs. self-sequences, but still showing that the spacers can also originate from the host chromosome.

The examples summarized above point to more-isolated alternative functions of CRISPR/Cas (rather than the main modus-operandi of the system). However, a question arises whether the involvement of the system in endogenous cellular processes (rather than the immune function) could be a major (or even the main) system function, at least for some CRISPR/Cas systems. It is interesting to investigate this question on the example of Type I-E CRISPR/Cas system in *E. coli* due to the following: (i) It is a classical, and best studied, Type I CRISPR/Cas system, (ii) *E. coli* is the host for the largest number of phage genomes (available in databases), and (iii) no alternative CRISPR/Cas functions have up-to-now been reported for this system. On the other hand, such involvement of the system in non-canonical functions seems plausible. In particular, interestingly, the spacer content of present-day Type I-E system of *E. coli* largely matches the *E. coli* CRISPR array sample, isolated from intestines of a 42,000-year-old mammoth [23]. This large time-scale certainly allows for an intensive spacer flow in response to suffered/undergone infections, which might suggest that the immune activity is not the prime modus-operandi of the aforementioned CRISPR/Cas system. Indeed, the native (in vivo) defense activity of the I-E system from *E. coli* has not been observed yet, so the maintenance of immunologically ineffective CRISPR/Cas system within the genome of *E. coli* might be promoted by the newly acquired functional modalities. It was also shown that cell envelope stress can activate CRISPR/Cas in *E. coli* and result in target interference [24], which might also indicate the involvement of this system in the endogenous regulatory processes (in addition to possibly being related with phage infection [25]).

Moreover, the possibility that CRISPR/Cas has a major involvement in gene expression regulation, also comes from another perspective, i.e., from biophysical modeling of Type I-E CRISPR/Cas dynamical response. Specifically, in our previous work, we showed that the main system regulatory features lead to the highly efficient expression of effector molecules (crRNAs), in a narrow time interval, with a specified time-delay corresponding to the signal onset [26,27,28,29]. It is clear that such highly efficient, and temporally specific response, may be highly desirable for gene expression regulation. In fact, we also previously showed that bacterial restriction modification systems—a more rudimental class of bacterial immune systems—exhibit gene expression regulation with such features [30,31,32].

Consequently, the possibility that Type I-E CRISPR/Cas dominantly exhibits non-canonical functions represents the main question that has been addressed in this paper. Such findings may contribute to the paradigm shift of CRISPR/Cas functioning, from a bacterial immune system, to a system that mainly regulates endogenous cellular processes. It would be very hard to directly experimentally address this question, mainly because it remains unclear under which conditions the native *E. coli* CRISPR/Cas is activated, i.e., even phage infection alone does not activate the system (i.e., under laboratory conditions the system is induced by artificially overexpressing Cas proteins and/or CRISPR array). That is, both CRISPR/Cas in *E. coli*, and its potentially regulated endogenous genes/pathways may be active only under specific (unknown) conditions, where possibly complex interplay between regulation of CRISPR/Cas and its targets may take place, which would be hard to assess experimentally. This question, however, can be more straightforwardly addressed through computational analysis. Regarding this, there are four potential CRISPR/Cas loci in *E. coli* genome [33]: Two of these have only one or two CRISPR spacers, one locus is a complete/independent CRISPR/Cas system of Type I-E (12 CRISPR spacers flanked by a full set of *cas* genes), while the remaining locus is a CRISPR array that lacks flanking *cas* genes. The complete Type I-E system is a standard model for CRISPR/Cas investigation, e.g., its transcription regulation has been comprehensively studied [27,34,35] and will be the subject of our analysis in this paper.

It is natural here to take a biophysical approach, i.e., to investigate interactions (approximated through sequence alignments) of CRISPR spacers with both chromosomal sequences and sequenced phage genomes. Another question is how to predict the putative function of the interactions with chromosomal sequence—are these interactions involved in the regulation of gene expression through protein-DNA interactions, or are the targets mRNA sequences (as in Type I-F case noted above). Namely, the available examples related to non-canonical CRISPR/Cas activity all point to endogenous mRNA targeting, followed by cleavage, as one of the most preferable mechanisms of the regulatory system functioning [15,16]. Clearly, the same mechanism with DNA targeting instead would result in autoimmunity. It has been shown, however, that the imperfect binding (i.e., mismatch-containing) of the interference machinery to its DNA targets does not result in cleavage, but rather in a sufficiently strong binding event [36,37]. DNA-targeting, therefore, also represents a possible avenue to impact the expression of regulated genes. Furthermore, if gene expression regulation is targeted, is it at the level of transcription regulation, or elongation (CRISPR complex presenting a roadblock for elongating RNA polymerase). Finally, what is the conservation of putative CRISPR targets, relative to the conservation of CRISPR spacers—i.e., is the rewiring of gene regulation by CRISPR/Cas limited by the turnover of target genome sequences (like in the regulation by transcription factors), or it is determined by more rapid turnover of CRISPR spacers (allowing abrupt changes of the entire regulon). We will address these questions below in the manuscript.

## 2. Materials and Methods

### 2.1. Sequence Datasets

Complete genome sequence and annotation of the *E. coli* str. K-12 substr. MG1655 was retrieved from the GenBank (NC_000913). Type I-E CRISPR array sequence and annotation of the corresponding strain was retrieved from the CRISPRdb [38]. Double-stranded DNA (dsDNA) bacteriophages, that infect *E. coli* (229 in total, accession numbers provided in the Supplementary Table S1), were assembled from Virus-Host DB (https://www.genome.jp/virushostdb/) and the corresponding genomic sequences with annotation were retrieved from the GenBank. For the obtained *E. coli* and phage genomes, 30 randomizations were created for the subsequent statistical analysis, with the maintenance of nucleotide content corresponding to individual coding and intergenic regions.

### 2.2. Sequence Alignment

To identify putative CRISPR spacer targets within *E. coli* and phage genomes, local pair-wise sequence alignment, based on Smith–Waterman algorithm [39], was done with ‘NUC44’ scoring matrix. For each genome, the alignment was performed against both direct and reverse strand, with 10 best hits reported, to enable the prediction of more than one putative spacer target per genomic strand. The obtained spacer alignments against all investigated genomes were arranged according to descending alignment scores, whereby the highest-scoring alignment of *E. coli* genome always corresponds to the spacer self-alignment located on the reverse strand (which is further excluded from the analysis, as being a trivial hit. The remaining alignments were grouped and further examined based on the following features: (i) Does the alignment fall within the coding vs. intergenic region; (ii) if within coding, is the putative spacer:hit interaction possible only at the level of gene DNA (‘RNA−’ category), or at the level of both gene DNA and mRNA sequence (‘RNA+’ category); (iii) if within intergenic, is the spacer target intergenic region categorized as divergent (putatively influencing both the upstream and downstream-encoded genes), convergent (with no putative influence on the neighboring genes) or upstream (putatively influencing only the downstream-encoded genes). Note that, for the previously defined categories, during the analysis of spacer alignment counts (SAC) and spacer alignment scores (SAS), we define the terms ‘SAC value’ and ‘SAS value’, which represent the sum of alignment counts and the average alignment scores for a given threshold, over the entire CRISPR array, respectively. For example, ‘SAC value’ for a threshold 3 was calculated from the first 3 alignments per spacer, as previously arranged.

### 2.3. Three-Nucleotide Motif Analysis

Analysis of putative Protospacer Adjacent Motifs (PAMs) related to the previously reported spacer alignments was conducted by examining 3nt-long segments flanking the theoretical start of the alignment, i.e., the first spacer nucleotide. Note that the actual alignment start does not necessarily match the first spacer coordinate. Consequently, the theoretical start of the alignment corresponds to the extension of the actual alignment start in the upstream direction, for the length corresponding to the unaligned portion of the CRISPR spacer. The appearance of every possible 3nt-long motif (64 in total) was counted at corresponding PAM position for every analyzed spacer alignment on the *E. coli* and randomized *E. coli* genomes. Additionally, due to the fact that the actual alignment start does not necessarily match the first spacer coordinate, 3nt-long motif analysis of sequences adjacent to real alignment start was performed: (i) 6 nucleotides upstream from the theoretical start of the alignment, and (ii) the segment between the theoretical and the actual start of the alignment.

### 2.4. Target Functional Enrichment Analysis

The functional annotation and the classification of putative targets were performed using the PANTHER classification system [40]. Only the hits (i.e., the corresponding proteins) associated with the experimentally confirmed PAMs [41] were subjected to this analysis. The search was conducted against the *E. coli* reference list for all annotation datasets, with statistics calculated according to Fisher’s Exact test, followed by an estimate of FDR (false discovery rate).

### 2.5. Conservation Analysis of CRISPR Spacers and Predicted Targets

All the investigated CRISPR spacers, along with predicted genomic targets (alignment hits) were subjected to a conservation analysis. CRISPR spacers were BLAST-ed against the non-redundant nucleotide database, with two search restrictions: (i) organism—bacteria; (ii) E-value < 10^−3^. The obtained BLAST hits were classified as a CRISPR array if they were surrounded by repeats, which was implemented by locally aligning 40nt long upstream and downstream flanking segments, with cutoff score of 25—a natural cut-off, below which the alignment score distribution decreases rapidly, characterized by a large number of mismatches in the alignment. CRISPR spacers and the sequences of predicted targets were also BLAST-ed (cutoff E-value of 10^−3^) against a representative NCBI set of prokaryotic genomes.

### 2.6. Statistical Analysis

The alignment score distributions were compared using independent t-test and Kolmogorov–Smirnov test (a nonparametric statistical test). Differentiation between the spacer alignments against the actual *E. coli* vs. randomized *E. coli* genomes, was done by normal distribution fitting and subsequent confidence bound estimation for the randomized genome hits, and further paired-sample t-test for determination of the statistical significance of the alignments on the *E. coli* genome. During PAM analysis the statistical over/underrepresentation of examined 3nt-long motifs was estimated by fitting the Poisson distribution to motif counts on randomized *E. coli* genomes to obtain corresponding confidence bounds, and subsequent p value calculation using paired-sample t-test. Estimations of statistically significant differences between phylogenetic distributions the Poisson distribution were used.

## 3. Results and Discussion

### 3.1. Type I-E CRISPR/Cas System of E. coli Preferentially Targets Endogenous Sequences

**Figure 1:**
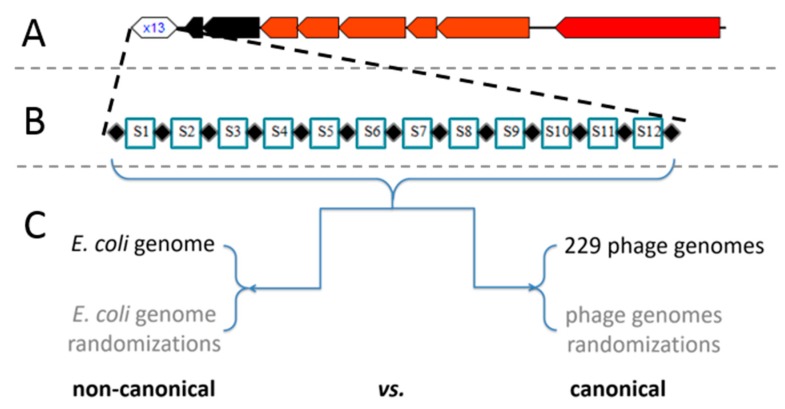
(**A**) Overall organization of the analyzed CRISPR/Cas locus generated by [42]. (**B**) Schematic organization of repeats and spacers within the CRISPR array. (**C**) Scheme of in silico analysis of canonical vs. non-canonical activity.

Our hypothesis is that the lack of spacer dynamics in the Type I-E CRISPR/Cas system of *E. coli* [4] is a possible consequence of the non-canonical system activity directed against endogenous sequences, whose role would be to regulate gene expression, as already observed for diverse CRISPR/Cas systems including I-F of *P. aeruginosa* [16]. Therefore, to differentiate between the canonical immune and this putative non-canonical, self-targeting, activity of the system, an extensive alignment analysis of the corresponding 12 CRISPR spacers against the *E. coli* genome and the genomes of 229 dsDNA bacteriophages that infect *E. coli* assembled from Virus-Host DB, was conducted (schematically shown in Figure 1, together with the overall organization of the CRISPR/Cas locus). If the self-targeting activity were the predominant system activity, then it would result in significantly higher alignment scores. Furthermore, to corroborate further obtained results, i.e., to determine the statistical significance of the reported hits, the analogous spacer alignment procedure was additionally conducted against 30 randomizations of every genomic sequence analyzed (for details see also Methods, Section 2.2 and Figure S1). In this way, the comparison was done both between the chromosome sequence and bacteriophage genomes, and also between the actual sequences and their randomized versions, which allows a robust estimate of the statistical significance for the obtained hits.

**Figure 2:**
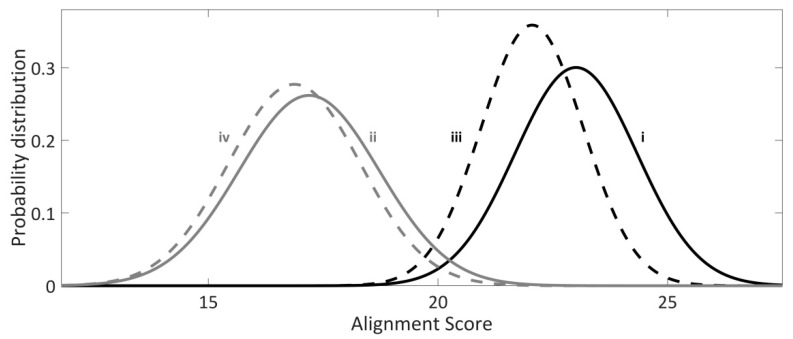
Normalized probability distributions of alignment scores for (**i**) *E. coli* genome—black solid curve, **(ii**) the genomes of all sequenced *E. coli* infecting bacteriophages—gray solid curve, (**iii**) randomized *E. coli* genome background—dashed black curve, (**iv**) randomized bacteriophage genome background—dashed grey curve.

In Figure 2, normalized distributions of the alignment scores for the hits reported within the genome of *E. coli* and the genomes of analyzed *E. coli*-infecting phages, as well for the corresponding genomic randomizations, are presented. Note that the alignment scores for the hits reported within the *E. coli* genome are significantly higher compared to the alignment scores of the hits reported within the randomized *E. coli* genomic sequences, as judged by the independent t-test and Kolmogorov–Smirnov test (*p* ~ 10^−36^, *p* ~ 10^−31^, respectively). On the contrary, according to the Kolmogorov–Smirnov test, no statistically significant difference (*p* = 0.19) is observed between the alignment scores of the hits reported within bacteriophage vs. randomized bacteriophage genomes, indicating that these hits could have arisen by chance. Additionally, alignment scores for the hits associated with the genome of *E. coli* are also significantly higher compared to the hits reported within the genomes of *E. coli*-infecting phages, according to both t-test and Kolmogorov–Smirnov test (*p* ≈ 0 for both tests), which, altogether indicates that Type I-E CRISPR/Cas system of *E. coli* preferentially targets endogenous sequences.

It is possible that some of the phages included in the analysis do not infect *E. coli*. However, CRISPR spacers are highly shared across the *E. coli* species, so that pieces of the attacker phage DNA would remain in CRISPR arrays of the present-day strains, even if ancestral strains had been attacked by the phages that no longer infect some of their descendants. A non-parametric Kolmogorov–Smirnov test was also used in the analysis, which is sensitive to the differences in the tails of the investigated distributions. Consequently, even if there were only some phages characterized by high-scoring hits/targets, these would be enriched in the distribution tail, and differentiated as significant with respect to the randomized background. Despite this, and contrary to *E. coli* targets, no statistical significance between real and randomized phage targets was observed.

The obtained result that Type I-E CRISPR/Cas system of *E. coli* preferentially targets endogenous sequences could in part be due to preferential adaptation from the bacterial chromosome [18]. On the other hand, the opposite trend reported in the literature—i.e., preferential sampling of spacers from foreign genetic material [22]—could indicate that those more rare sequences, which end up integrated into CRISPR array from bacterial/host chromosome, are under positive selection to be kept within the array, which would indicate that corresponding interactions with endogenous targets are indeed functional. To further assess the functional significance of this preferential targeting, we next analyze in depth the predicted targets in genome sequences.

### 3.2. CRISPR Spacer and Target Sequence Conservation Analysis

**Figure 3:**
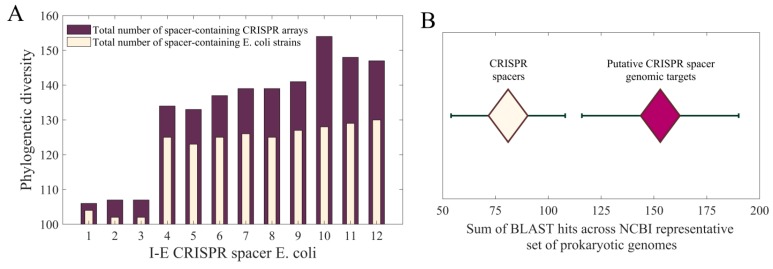
(**A**) Phylogenetic distribution of I-E *E. coli* CRISPR spacers throughout the domain of bacteria vs. *E. coli* species; (**B**) Phylogenetic distribution of I-E *E. coli* CRISPR spacers and their putative genomic targets across the NCBI representative set of prokaryotic genomes. For CRISPR array, the sum of hits obtained for all 12 spacers (with three standard deviations) is shown (white diamond). For CRISPR spacer genomic targets (19 targets per spacer), the mean number of hits, summed over 12 spacers, with three standard deviations, is shown (purple diamond).

Two-fold conservation analysis of the Type I-E *E. coli* CRISPR spacers was performed (i) across the domain of bacteria; (ii) across the completed genomes of *E. coli* species only. In the Figure 3A, in both cases, the trend of an increasing number of spacer-containing species from ‘youngest’ to ‘oldest’ spacer (S1–S12, consecutively) was observed, suggesting that the I-E CRISPR/Cas system was ‘actively’ incorporating spacers in the past. Note that the overall difference in the number of reported hits between these two categories is caused by the fact that in the first category the hits are also associated with partial genomic sequences and scaffolds, where, interestingly, only the spacer 10 contains hits outside the *Escherichia* genus. The hits associated with the spacer 10 also appear in the closely related *Shigella* genus, so that the observed trend of limited phylogenetic distribution of the investigated CRISPR array remains robust.

In addition, the phylogenetic distribution of CRISPR spacers and their putative genomic targets were inferred across the NCBI representative set of prokaryotic genomes (Figure 3B). The statistically significant difference, at the level of 3σ, between the number of reported hits was observed, where higher values appear associated with the investigated targets of CRISPR spacers. As the conservation of the *cis*-regulated sites appears higher compared to the conservation of their *trans*-acting factors, the functionality of the former (i.e., putative CRISPR spacer genomic targets) could not be confirmed through the standard phylogenetic footprinting analysis.

The observed gradient in spacer conservation might be important in its own right, since no gradient in spacer transcription was found (i.e., all spacers are expressed at the same level [27]). That is, the canonical system function would presumably be more consistent with older and phylogenetically more widespread spacers (that might correspond to remnants of past infections) being less active than young spacers—moreover, this as a CRISPR precursor transcript contains multiple palindromic repeats that might function as transcription terminators [27]. The finding that putative targets (*cis*-regulated sites) are more conserved compared to CRISPR spacers (their *trans*-acting factors) has important implications related to the putative mode of regulatory endogenous activity by CRISPR/Cas systems. Namely, standard *trans*-factors (protein-coding transcription factors) are much more conserved compared to their genomic *cis*-regulatory elements — consequently, it is the turnover of the *cis*-regulatory elements that determines how fast regulatory interactions can be rewired. Here we found an opposite regime, where the turn-over rate of *cis*-regulatory elements (CRISPR spacer targets) is lower compared to their *trans*-factors (CRISPR spacers), so that CRISPR spacers now determine the rewiring rate of a regulatory circuit. Consequently, while in the standard case (protein transcription factors), the changes in regulatory network topology are determined by gain/loss of the individual *cis*-regulatory sites, for CRISPR/Cas systems this change is determined by gain or loss of CRISPR spacers—i.e., of the entire *trans*-factor, which can abruptly perturb a number of regulatory interactions.

### 3.3. Target Preference for Coding vs. Intergenic Regions

As observed in the previous section, statistically significant, higher alignment scores characterize only *E. coli* genomic hits, compared to the other investigated systems (*E. coli*-infecting phage genomes, randomized *E. coli* genomes). We next wanted to infer if there are any functional criteria that would enable further differentiation of these hits and, therefore, point to putative targeting preferences of the interference machinery. To achieve this, obtained *E. coli* hits were systematized according to a previously devised set of criteria (see Methods, Section 2.2) and associated alignment characteristics were compared. To portray the underlying alignment differences, two complementary measures were defined, SAC (spacer alignment counts) and SAS (spacer average alignment scores) values (see Methods, Section 2.2).

**Figure 4:**
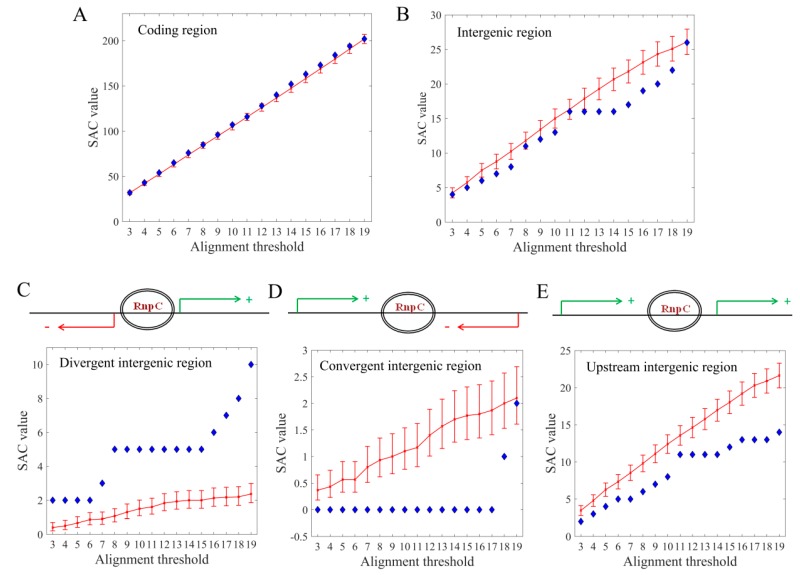
(**A**) SAC values associated with hits located in coding and (**B**) intergenic regions of *E. coli* vs. randomized *E. coli* background; (**C**) SAC values associated with hits located in divergent, (**D**) convergent and (**E**) upstream intergenic regions, preceded by schematic representation of the intergenic region classification; On each plot, SAC values are shown as a function of the growing number of hits per spacer, with confidence bound estimates provided for the SAC values associated with randomized background.

To infer the possible mechanism of endogenous CRISPR/Cas I-E activity, the affinity of the interference machinery towards coding vs. non-coding (intergenic) sequences was first investigated. In Figure 4A,B and Figure S2, no preference of the interference machinery towards intergenic regions can be found—in fact, for higher thresholds there is a somewhat smaller preference towards the intergenic regions. To further dissect this preference, intergenic regions were divided into three categories: divergent, upstream, and convergent, based on the orientation of the neighboring genes. Namely, intergenic regions that are upstream of both of the neighboring genes are classified as divergent. Therefore, regulatory interactions accomplished in these regions, could interfere with transcription initiation of both flanking genes/operons. Similarly, intergenic regions that are upstream of only one of the neighboring genes are classified as upstream. In this case, transcription initiation of only one particular gene/operon can be regulated by the interference machinery. Finally, intergenic regions that are downstream of both of the adjacent genes are classified as convergent, so that no influence is feasible on the transcription initiation of these neighboring genes. Whereas any preferences of the interference machinery towards intergenic regions, in general, have not been observed, this detailed analysis (Figure 4C) enabled us to detect that the number of alignments (i.e., the corresponding SAC value) from the divergent category, is significantly higher compared to the average SAC value in the dataset with randomized *E. coli* genomes, over a broad threshold range (3–19). On the contrary, the SAC value of the hits from the convergent intergenic regions (Figure 4D), where the binding of the interference machinery is ineffective in terms of any regulatory activities, is drastically underrepresented compared to the randomized background, again, over a broad threshold range (this drastic underrepresentation also leads to the absence of preference for total intergenic regions). Finally, in Figure 4E one can observe that the SAC values associated with alignments from the upstream intergenic regions are slightly below the randomized background, which is in accordance with only a moderate, putative impact on the transcription initiation of the neighboring genes/operons.

### 3.4. Target Preference for DNA vs. RNA

**Figure 5:**
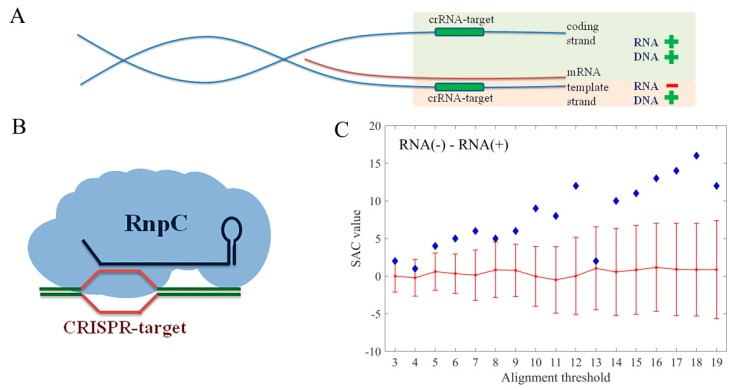
(**A**) Schematic representation of the functional classification of hits associated with coding regions, based on the DNA strand that is being targeted, and (**B**) the interference RnpC; (**C**) The plot showing the difference between SAC values associated with targets classified as RNA− and RNA+ category.

As discussed above, another important issue is whether RnpC has the preference to target DNA or RNA sequences. To that end, the hits associated with the *E. coli* coding regions were further divided to RNA+ and RNA− categories, based on the strand that was predicted to be targeted by the interference machinery (Figure 5A,B). Note that, if a spacer hit was reported on one DNA strand, the real target is located on its reverse complement, since crRNA, that is complementary to the analyzed spacer, is part of the interference complex that recognizes the target. Therefore, if the predicted hit was located on the coding strand of a gene, the actual target is on the template strand, so that the interference machinery can influence gene expression only at the level of DNA. Consequently, these hits were denoted as RNA−. Vice versa, if the hit was predicted on the template strand of a gene, the actual target of the crRNA is located on the coding strand, so that the interference machinery can influence gene expression at the level of both DNA and mRNA. These hits were, therefore, denoted as RNA+. In Figure 5C a clear and statistically significant preference of the interference machinery towards RNA− category was observed, indicating that the regulatory action of the *E. coli* I-E system preferentially targets the transcription of the regulated gene. In summary, when targeting the coding regions, the interference machinery prefers binding to the template strand of a gene, that is, it preferentially targets the DNA itself. Likewise, among the binding events hitting non-coding sequences, the regulatory ones are the most effective, i.e., divergent-contained, appear as statistically overrepresented.

To summarize the results of the previous two subsections, those genomic regions that enable the most efficient interference with the transcription of associated genes, i.e., divergent intergenic and RNA− coding regions, appear as statistically highly overrepresented. For upstream intergenic regions, that are able to impact only the transcription of downstream genes, moderate statistical underrepresentation is also observed. Note, however, that this specific category comprises a very large fraction of total intergenic regions, so the full number of targets associated with this category is not small, despite the observed overall underrepresentation compared to the randomized background. Importantly, in a case where the interference machinery cannot influence the transcription of the neighboring genes (for the convergent intergenic regions), practically no hits are observed. Taken together, these results imply that the heterogeneous pattern of targets is accepted as long as it enables the interference machinery to act as efficient as possible at the level of transcription. This interference with the first step of gene expression is obviously energetically very favorable for a bacterial cell, and, hence, probably recognized and accepted as a most-preferable model for the non-canonical activity of the Type I-E CRISPR/Cas system of *E. coli*.

### 3.5. Target Strand Bias

**Figure 6:**
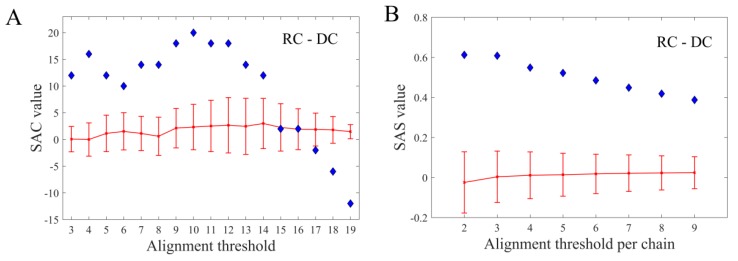
(**A**) The plot showing the difference between SAC and (**B**) SAS values associated with hits on the reverse and direct DNA strand of the *E. coli* genome shown as a function of the increasing number of hits per chain.

The results presented thus far strongly imply the self-targeting activity of the *E. coli* CRISPR/Cas I-E system, so another important aspect to elucidate was the genomic origin of spacers. Therefore, analysis of the CRISPR-target topology across the genome of *E. coli* was performed. Note here that, besides the association of CRISPR-adaptation events with the Chi and Ter sites, there is no clear indication about the relatedness between distinguished genomic features and the spacer selection process so far. In line with this, only the basic analysis, that accounts for preference of the interference machinery towards a certain genomic strand, was conducted. The SAC values from Figure 6A indicate that the interference machinery clearly favors the reverse, over the direct strand (RC and DC). Additionally, the SAS analysis (Figure 6B) reveals that the scores (SAS values) associated with the RC are significantly higher compared to the ones associated with the DC, where this statistically significant difference was observed when comparing alignments from the randomized background. This, altogether, implies that the spacer selection apparently co-occurs with a strand-sensitive process, operating on a genome-scale.

Consequently, the results above suggest that the spacers are integrated into the CRISPR array in a strand-sensitive process, which operates on a genome scale. A plausible explanation is the association with DNA replication, because during this process DNA polymerase makes a clear difference between the leading and the lagging DNA strand. It should also be noted that the by-products of DNA replication, which arises with the reparation of stalled replication forks [20,22], may act as substrates for Cas1/Cas2 complex and be integrated into the CRISPR array during the process of naïve adaptation [18,19,20].

### 3.6. PAM Analysis

**Figure 7:**
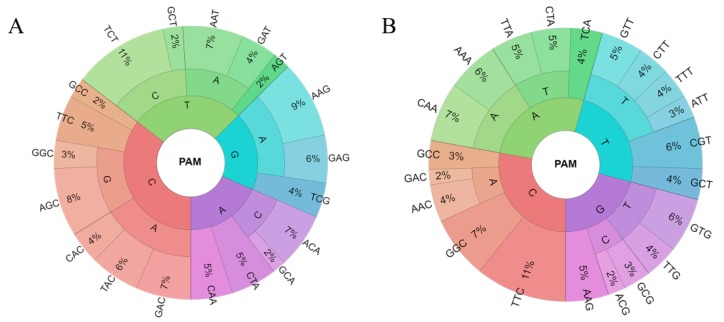
(**A**) The PAM-wheel of 3nt-long statistically overrepresented motifs flanking the theoretical and (**B**) the actual start of the alignment on the targeted strand, in the 3′–5′ direction. The percentages are shown as a function of the associated Z scores [(actual motif counts—sum of all overrepresented motif counts)/sum of all overrepresented motif counts)].

To further corroborate the functionality of the predicted targets, overrepresentation analysis of the 3nt-long motifs was conducted with the aim of identifying PAMs associated with targeted sequences. The relative abundance of the overrepresented motifs associated with PAM-position next to the theoretical start of the alignment and a wider region, flanking the real start of the alignment (for more details see Methods, Section 2.3) was shown as a PAM-wheel (Figure 7A,B). These motifs were further compared against the experimentally confirmed interference PAMs [41], where the first set of motifs (Figure 7A) shows the robustness of PAM-percentage overlap (the constant of ~30%), for the investigated alignment thresholds (3–10). On the contrary, the second set of motifs (Figure 7B) shows an increase in PAM-overlap from 33% to 50%, over the same threshold range.

The trend of increase noted above could be a consequence of decreasing binding affinity between RnpC and predicted targets, as assessed by the alignment score, which is compensated by an increased affinity between the same machinery and PAM(s). Namely, the accumulation of PAMs right upstream of the alignment enables sufficiently strong anchoring of the RnpC, to compensate for weaker interactions with the target in the course of R-loop formation. This mechanism of mutual complementation, known as a mix-and-match model, has already been found for transcription initiation, i.e., was first proposed for bacterial σ^70^ factors [43,44], and subsequently also extended by us to the case of alternative sigma factors [45,46].

### 3.7. Functional Enrichment Analysis

In order to identify the metabolic processes preferentially targeted by the interference machinery, functional enrichment analysis was further conducted for the hits for the threshold 7 (which appeared as a natural cut-off in the analysis, see Figure 4C). All the proteins, which are predicted to be targeted by the RnpC and appeared associated with experimentally determined interference PAMs (with respect to the theoretical start of the alignment), were subjected to Panther search against Gene Ontology database. The analysis against all annotation datasets was supplemented with estimation of statistical significance. The statistically significant annotations were obtained against PANTHER GO-Slim Biological Process and GO biological process complete datasets (for results see SI_Panther_analysis), where transposition and DNA recombination emerged as the most specific functional annotations.

Statistically significant enrichment of transposition and DNA recombination pathways implies that despite fundamental differences between non-canonical and canonical means of action (i.e., targeting self vs. non-self), Type I-E CRISPR/Cas system of *E. coli* in both cases may keep its core function to regulate the horizontal gene transfer. A specific type of prokaryotic self-synthesizing transposons, named casposons, have also been previously proposed by Krupovic et al. as a unique origin of Class I CRISPR/Cas adaptation modules [47], where Cas1 and casposase—the main integration enzyme of casposons, share mechanistic similarities even at the level of specificity for DNA [48]. This may establish a link between Type I CRISPR/Cas system and transposition pathways, which may stand behind the functional enrichment analysis result. Finally, for the Type I-A system of *Sulfolobus islandicus* a regulatory (transcriptional) link between the spacer acquisition and DNA recombination genes has been experimentally shown [49], which also aligns with the predicted enrichment of DNA recombination pathway to putatively be influenced by the Type I-E CRISPR/Cas system of *E. coli*.

## 4. Conclusions

We found that spacers of Type I-E CRISPR/Cas system in *E. coli* have a highly pronounced tendency to target the host over bacteriophage genomes. It is sometimes noted that the representation of phage genomic sequences across databases does not fully reflect their diversity in the environment. However, this is less likely to be an obstacle in this case, as the number of sequenced phage genomes, used in this study, is very large (>200). As an additional control, the distribution of the alignment scores for the host genome highly deviates from the random ensemble, which is not the case for phage genomes. The distribution of the hits in the host genome is highly non-random: It is strand-specific (highly preferring reverse over direct genome strand) and has a clear preference for regions related with transcription or its regulation (the hits are severely underrepresented in the convergent intergenic regions, where no transcription or its regulation is expected, while overrepresented in the divergent intergenic regions that should regulate both of the adjacent genes). Moreover, hits have clear DNA strand orientation, i.e., binding to DNA strands which could influence/target mRNA is clearly disfavored. Overall these results strongly suggest that Type I-E CRISPR/Cas in *E. coli* primarily targets sequences in bacterial chromosome, rather than foreign DNA invaders. Further, the regulation appears to happen at the level of transcription regulation, rather than at the level of mRNA degradation, in line with the previous results that target sequences with mismatches will not be cut by RnpC, but that RnpC will bind to them, likely interfering with transcription. Since the targets do not distinguish coding from intergenic regions, we hypothesize that this interference might be exhibited both at the level of transcription initiation (when the binding happens at, or close to, the upstream intergenic regions), and at the level of transcription elongation (when RnpC binds to the coding region, presenting a roadblock for elongating RNA polymerase [32]).

Based on these results, we propose the following hypothesis for spacer acquisition in Type I-E CRISPR/Cas, which puts them under unified framework: Substrates for naïve adaptation may be generated from *E. coli* genome through processes such as transcription and replication. This may already create some bias in the sequences integrated into the CRISPR array, and consequently also to their targets, as naïve adaptation may prefer sequences that are actively transcribed or preferentially coming from one DNA strand (as the replication is strand-specific). Furthermore, the targets of the newly integrated spacer have to provide a selective advantage (or at least not to interfere) with the system function, for the newly acquired spacer to be retained. This may effectively couple the selection of CRISPR spacers to a set of functional targets on genome sequence—mutations in the bacterial genome that lead to sequences that can beneficially interact with RnpC of the new spacer, will also likely be stabilized by positive selection. Overall, this may lead to the target pattern in the host genome that is highly non-random, as observed in our study.

As an outlook, the strong indication that the classical (model) CRISPR/Cas system in *E. coli* is dominantly involved in regulation of endogenous gene expression, rather than in system immune response, provided by this study, adds to a growing body of evidence on the importance of non-canonical functions in CRISPR/Cas. Investigating such functions is coupled with significant difficulties from both the experimental and computational side, mainly related to the fact that CRISPR/Cas is typically not active under standard conditions. Moreover, in Type II systems (and possibly in other types) there is also significant difficulty in identifying system components related with non-canonical functions, which are mainly connected with still low availability of RNA-seq data across diverse bacterial strains, and also generally low conservation of small RNAs across bacterial genomes—see [17] and references therein. Despite the difficulties noted above, the effort is highly worthwhile in terms of both understanding basic CRISPR/Cas biology and biotechnology applications. In particular, the detailed in silico analysis done here for Type I-E system in *E. coli* should be extended to diverse CRISPR/Cas systems in other bacterial systems, or even to metagenomic datasets, where tools well suited for such analysis have been developed [50]. Such computational studies should provide a rational starting point for further experiments, where stand-alone computational studies proved to be important for understanding CRISPR/Cas systems [8,12,51]. Consequently, a synergy between experimental and computational studies may lead to a much better characterization of non-canonical functions in CRISPR/Cas systems.

## Supplementary Materials

The following are available online, Figure S1: Z value expressed as a function of the number of alignments, Figure S2: SAC value expressed as a function of the number of alignments, Table S1: List of *E. coli*-infecting dsDNA bacteriophages with accession numbers. SI_Panther_analysis.

## References

[1] Kirchner M., Schneider S. (2015). CRISPR-Cas: From the Bacterial Adaptive Immune System to a Versatile Tool for Genome Engineering. Angew. Chem. Int. Ed..

[2] Barrangou R., Marraffini L.A. (2014). CRISPR-Cas systems: Prokaryotes upgrade to adaptive immunity. Mol. Cell.

[3] Caplan A.L., Parent B., Shen M., Plunkett C. (2015). No time to waste—the ethical challenges created by CRISPR: CRISPR/Cas, being an efficient, simple, and cheap technology to edit the genome of any organism, raises many ethical and regulatory issues beyond the use to manipulate human germ line cells. EMBO Rep..

[4] Vora S., Tuttle M., Cheng J., Church G. (2016). Next stop for the CRISPR revolution: RNA-guided epigenetic regulators. FEBS J..

[5] Cano-Rodriguez D., Rots M.G. (2016). Epigenetic editing: On the verge of reprogramming gene expression at will. Curr. Genet. Med. Rep..

[6] Choi K.R., Lee S.Y. (2016). CRISPR technologies for bacterial systems: Current achievements and future directions. Biotechnol. Adv..

[7] Li X.T., Jun Y., Erickstad M.J., Brown S.D., Parks A., Court D.L., Jun S. (2016). tCRISPRi: Tunable and reversible, one-step control of gene expression. Sci. Rep..

[8] Makarova K.S., Grishin N.V., Shabalina S.A., Wolf Y.I., Koonin E.V. (2006). A putative RNA-interference-based immune system in prokaryotes: Computational analysis of the predicted enzymatic machinery, functional analogies with eukaryotic RNAi, and hypothetical mechanisms of action. Biol. Direct.

[9] Barrangou R., Fremaux C., Deveau H., Richards M., Boyaval P., Moineau S., Romero D.A., Horvath P. (2007). CRISPR provides acquired resistance against viruses in prokaryotes. Science.

[10] Yosef I., Goren M.G., Qimron U. (2012). Proteins and DNA elements essential for the CRISPR adaptation process in Escherichia coli. Nucleic Acids Res..

[11] Datsenko K.A., Pougach K., Tikhonov A., Wanner B.L., Severinov K., Semenova E. (2012). Molecular memory of prior infections activates the CRISPR/Cas adaptive bacterial immunity system. Nat. Commun..

[12] Bolotin A., Quinquis B., Sorokin A., Ehrlich S.D. (2005). Clustered regularly interspaced short palindrome repeats (CRISPRs) have spacers of extrachromosomal origin. Microbiology.

[13] Gunderson F.F., Mallama C.A., Fairbairn S.G., Cianciotto N.P. (2015). Nuclease activity of Legionella pneumophila Cas2 promotes intracellular infection of amoebal host cells. Infect. Immun..

[14] Louwen R., Horst-Kreft D., de Boer A.G., van der Graaf L., de Knegt G., Hamersma M., Heikema A.P., Timms A.R., Jacobs B.C., Wagenaar J.A. (2013). A novel link between Campylobacter jejuni bacteriophage defence, virulence and Guillain-Barre syndrome. Eur. J. Clin. Microbiol. Infect. Dis..

[15] Sampson T.R., Saroj S.D., Llewellyn A.C., Tzeng Y.L., Weiss D.S. (2013). A CRISPR/Cas system mediates bacterial innate immune evasion and virulence. Nature.

[16] Li R., Fang L., Tan S., Yu M., Li X., He S., Wei Y., Li G., Jiang J., Wu M. (2016). Type I CRISPR-Cas targets endogenous genes and regulates virulence to evade mammalian host immunity. Cell Res..

[17] Guzina J., Chen W.-H., Stankovic T., Djordjevic M., Zdobnov E., Djordjevic M. (2018). In-silico analysis suggests common appearance of scaRNAs in Type II systems and their association with bacterial virulence. Front. Genet..

[18] Radovcic M., Killelea T., Savitskaya E., Wettstein L., Bolt E.L., Ivancic-Bace I. (2018). CRISPR-Cas adaptation in Escherichia coli requires RecBCD helicase but not nuclease activity, is independent of homologous recombination, and is antagonized by 5’ ssDNA exonucleases. Nucleic Acids Res..

[19] Ivancic-Bace I., Cass S.D., Wearne S.J., Bolt E.L. (2015). Different genome stability proteins underpin primed and naive adaptation in E. coli CRISPR-Cas immunity. Nucleic Acids Res..

[20] Shiimori M., Garrett S.C., Chambers D.P., Glover C.V.C., Graveley B.R., Terns M.P. (2017). Role of free DNA ends and protospacer adjacent motifs for CRISPR DNA uptake in Pyrococcus furiosus. Nucleic Acids Res..

[21] Musharova O., Vyhovskyi D., Medvedeva S., Guzina J., Zhitnyuk Y., Djordjevic M., Severinov K., Savitskaya E. (2018). Avoidance of Trinucleotide Corresponding to Consensus Protospacer Adjacent Motif Controls the Efficiency of Prespacer Selection during Primed Adaptation. mBio.

[22] Levy A., Goren M.G., Yosef I., Auster O., Manor M., Amitai G., Edgar R., Qimron U., Sorek R. (2015). CRISPR adaptation biases explain preference for acquisition of foreign DNA. Nature.

[23] Savitskaya E., Lopatina A., Medvedeva S., Kapustin M., Shmakov S., Tikhonov A., Artamonova I.I., Logacheva M., Severinov K. (2017). Dynamics of Escherichia coli type I-E CRISPR spacers over 42,000 years. Mol. Ecol..

[24] Perez-Rodriguez R., Haitjema C., Huang Q., Nam K.H., Bernardis S., Ke A., DeLisa M.P. (2011). Envelope stress is a trigger of CRISPR RNA-mediated DNA silencing in Escherichia coli. Mol. Microbiol..

[25] Imamovic L., Martinez-Castillo A., Benavides C., Muniesa M. (2015). BaeSR, involved in envelope stress response, protects against lysogenic conversion by Shiga toxin 2-encoding phages. Infect. Immun..

[26] Rodic A., Blagojevic B., Djordjevic M., Severinov K., Djordjevic M. (2017). Features of CRISPR-Cas regulation key to highly efficient and temporally-specific crRNA production. Front. Microbiol..

[27] Pougach K., Semenova E., Bogdanova E., Datsenko K.A., Djordjevic M., Wanner B.L., Severinov K. (2010). Transcription, processing and function of CRISPR cassettes in Escherichia coli. Mol. Microbiol..

[28] Djordjevic M., Djordjevic M., Severinov K. (2012). CRISPR transcript processing: A mechanism for generating a large number of small interfering RNAs. Biol. Direct.

[29] Rodic A., Blagojevic B., Djordjevic M. (2018). Systems Biology of Bacterial Immune Systems: Regulation of Restriction-Modification and CRISPR-Cas Systems. Systems Biology.

[30] Rodic A., Blagojevic B., Zdobnov E., Djordjevic M., Djordjevic M. (2017). Understanding key features of bacterial restriction-modification systems through quantitative modeling. BMC Syst. Biol..

[31] Morozova N., Sabantsev A., Bogdanova E., Fedorova Y., Maikova A., Vedyaykin A., Rodic A., Djordjevic M., Khodorkovskii M., Severinov K. (2016). Temporal dynamics of methyltransferase and restriction endonuclease accumulation in individual cells after introducing a restriction-modification system. Nucleic Acids Res..

[32] Klimuk E., Bogdanova E., Nagornykh M., Rodic A., Djordjevic M., Medvedeva S., Pavlova O., Severinov K. (2018). Controller protein of restriction-modification system Kpn2I affects transcription of its gene by acting as a transcription elongation roadblock. Nucleic Acids Res..

[33] Diez-Villasenor C., Almendros C., Garcia-Martinez J., Mojica F.J. (2010). Diversity of CRISPR loci in Escherichia coli. Microbiology.

[34] Pul U., Wurm R., Arslan Z., Geissen R., Hofmann N., Wagner R. (2010). Identification and characterization of *E. coli* CRISPR-cas promoters and their silencing by H-NS. Mol. Microbiol..

[35] Westra E.R., Pul U., Heidrich N., Jore M.M., Lundgren M., Stratmann T., Wurm R., Raine A., Mescher M., Van Heereveld L. (2010). H-NS-mediated repression of CRISPR-based immunity in Escherichia coli K12 can be relieved by the transcription activator LeuO. Mol. Microbiol..

[36] Jinek M., Chylinski K., Fonfara I., Hauer M., Doudna J.A., Charpentier E. (2012). A programmable dual-RNA-guided DNA endonuclease in adaptive bacterial immunity. Science.

[37] Sternberg S.H., LaFrance B., Kaplan M., Doudna J.A. (2015). Conformational control of DNA target cleavage by CRISPR-Cas9. Nature.

[38] Grissa I., Vergnaud G., Pourcel C. (2007). CRISPRFinder: A web tool to identify clustered regularly interspaced short palindromic repeats. Nucleic Acids Res..

[39] Barton G.J. (1993). An efficient algorithm to locate all locally optimal alignments between two sequences allowing for gaps. Comput. Appl. Biosci..

[40] Mi H., Huang X., Muruganujan A., Tang H., Mills C., Kang D., Thomas P.D. (2017). PANTHER version 11: Expanded annotation data from Gene Ontology and Reactome pathways, and data analysis tool enhancements. Nucleic Acids Res..

[41] Fu B.X., Wainberg M., Kundaje A., Fire A.Z. (2017). High-Throughput Characterization of Cascade type I-E CRISPR Guide Efficacy Reveals Unexpected PAM Diversity and Target Sequence Preferences. Genetics.

[42] Zhang Q., Ye Y. (2017). Not all predicted CRISPR-Cas systems are equal: Isolated cas genes and classes of CRISPR like elements. BMC Bioinform..

[43] Hook-Barnard I.G., Hinton D.M. (2007). Transcription initiation by mix and match elements: Flexibility for polymerase binding to bacterial promoters. Gene Regul. Syst. Biol..

[44] Djordjevic M. (2011). Redefining Escherichia coli sigma(70) promoter elements: -15 motif as a complement of the -10 motif. J. Bacteriol..

[45] Guzina J., Djordjevic M. (2016). Promoter recognition by ECF sigma factors: Analyzing DNA and protein interaction motifs. J. Bacteriol..

[46] Guzina J., Djordjevic M. (2017). Mix-and-matching as a promoter recognition mechanism by ECF sigma factors. BMC Evol. Biol..

[47] Krupovic M., Makarova K.S., Forterre P., Prangishvili D., Koonin E.V. (2014). Casposons: A new superfamily of self-synthesizing DNA transposons at the origin of prokaryotic CRISPR-Cas immunity. BMC Biol..

[48] Beguin P., Charpin N., Koonin E.V., Forterre P., Krupovic M. (2016). Casposon integration shows strong target site preference and recapitulates protospacer integration by CRISPR-Cas systems. Nucleic Acids Res..

[49] Liu T., Liu Z., Ye Q., Pan S., Wang X., Li Y., Peng W., Liang Y., She Q., Peng N. (2017). Coupling transcriptional activation of CRISPR-Cas system and DNA repair genes by Csa3a in Sulfolobus islandicus. Nucleic Acids Res..

[50] Sharifi F., Ye Y. (2017). From Gene Annotation to Function Prediction for Metagenomics. Methods Mol. Biol..

[51] Mojica F.J., Diez-Villasenor C., Garcia-Martinez J., Soria E. (2005). Intervening sequences of regularly spaced prokaryotic repeats derive from foreign genetic elements. J. Mol. Evol..

