# Supplementary material for: Endogenous Gene Regulation as a Predicted Main Function of Type I-E CRISPR/Cas System in E. coli
Source: Molecules. 2019 Feb 21;24(4):784. doi: 10.3390/molecules24040784 (PMC6413058; doi:10.3390/molecules24040784)
Supplement: Supplementary file 1 [file molecules-24-00784-s001.zip › molecules-429134-SM-1.pdf]

Endogenous gene regulation as a predicted main function of Type I-E CRISPR/Cas system in *E. coli*

Bojan Đ. Božić, Jelena Repac, Marko Đorđević*
Institute of Physiology and Biochemistry, Faculty of Biology, University of Belgrade, Studentski trg 16, 11000 Belgrade, Serbia
*Corresponding author. Tel.: +381 11 3033356; e-mail: dmarko@bio.bg.ac.rs

Table S1. List of *E. coli*-infecting dsDNA bacteriophages with accesion numbers.

**Bacteriophage**	**Accesion number**
Enterobacter phage CC31 [TAX:709484]	NC_014662
Enterobacteria phage 13a [TAX:532076]	NC_011045
Enterobacteria phage 2851 [TAX:254397]	FM180578
Enterobacteria phage 285P [TAX:669008]	NC_015249
Enterobacteria phage 933W [TAX:10730]	NC_000924
Enterobacteria phage 9g [TAX:1468411]	NC_024146
Enterobacteria phage BA14 [TAX:532074]	NC_011040
Enterobacteria phage BP-4795 [TAX:196242]	NC_004813
Enterobacteria phage Bp7 [TAX:1052121]	NC_019500
Enterobacteria phage cdtI [TAX:414970]	NC_009514
Enterobacteria phage CUS-3 [TAX:539221]	CP000711
Enterobacteria phage DE3 [TAX:482822]	EU078592
Enterobacteria phage DT571/2 [TAX:1567007]	KM979355
Enterobacteria phage EcoDS1 [TAX:532075]	NC_011042
Enterobacteria phage fiAA91-ss [TAX:1357825]	NC_022750
Enterobacteria phage GEC-3S [TAX:1222338]	NC_025425
Enterobacteria phage HK106 [TAX:432198]	NC_019768
Enterobacteria phage HK140 [TAX:1147143]	NC_019710
Enterobacteria phage HK225 [TAX:1147144]	NC_019717
Enterobacteria phage HK446 [TAX:1147145]	NC_019714
Enterobacteria phage HK542 [TAX:432200]	NC_019769
Enterobacteria phage HK544 [TAX:432201]	NC_019767
Enterobacteria phage HK629 [TAX:1147148]	NC_019711
Enterobacteria phage HK630 [TAX:1147146]	NC_019723
Enterobacteria phage HK633 [TAX:1147147]	NC_019719
Enterobacteria phage IME08 [TAX:697227]	NC_014260
Enterobacteria phage IME10 [TAX:1090133]	NC_019501
Enterobacteria phage J8-65 [TAX:1536597]	NC_025445
Enterobacteria phage JenK1 [TAX:1610836]	NC_029021

Enterobacteria phage JenP1 [TAX:1610837]	NC_029028
Enterobacteria phage JenP2 [TAX:1610838]	NC_028997
Enterobacteria phage JS10 [TAX:576790]	NC_012741
Enterobacteria phage K1F [TAX:344021]	NC_007456
Enterobacteria phage mEp043 c-1 [TAX:1147149]	NC_019706
Enterobacteria phage mEp235 [TAX:1147150]	NC_019708
Enterobacteria phage mEp237 [TAX:1147151]	NC_019704
Enterobacteria phage mEp460 [TAX:1147152]	NC_019716
Enterobacteria phage mEpX1 [TAX:1147153]	NC_019709
Enterobacteria phage mEpX2 [TAX:1147154]	NC_019705
Enterobacteria phage P7 [TAX:10682]	AF503408
Enterobacteria phage P88 [TAX:1567486]	NC_026014
Enterobacteria phage Phi1 [TAX:448384]	NC_009821
Enterobacteria phage phi80 [TAX:10713]	NC_021190
Enterobacteria phage phi92 [TAX:948870]	NC_023693
Enterobacteria phage phiEcoM-GJ1 [TAX:451705]	NC_010106
Enterobacteria phage phiJLA23 [TAX:1273706]	KC333879
Enterobacteria phage phiP27 [TAX:103807]	NC_003356
Enterobacteria phage RB10 [TAX:69608]	KM606999
Enterobacteria phage RB27 [TAX:69609]	NC_025448
Enterobacteria phage RB33 [TAX:134822]	KM607001
Enterobacteria phage RB49 [TAX:50948]	NC_005066
Enterobacteria phage RB5 [TAX:1112578]	KM606995
Enterobacteria phage RB51 [TAX:10693]	NC_012635
Enterobacteria phage RB55 [TAX:697289]	KM607002
Enterobacteria phage RB59 [TAX:697290]	KM607003
Enterobacteria phage RB6 [TAX:69610]	KM606996
Enterobacteria phage RB68 [TAX:36339]	NC_027979
Enterobacteria phage RB69 [TAX:12353]	NC_004928
Enterobacteria phage RB7 [TAX:697291]	KM606997
Enterobacteria phage RB9 [TAX:69612]	KM606998
Enterobacteria phage T3 [TAX:10759]	NC_003298
Enterobacteria phage T4T [TAX:857277]	HM137666
Enterobacteria phage T7 [TAX:10760]	NC_001604
Enterobacteria phage T7M [TAX:1075774]	JX421753
Enterobacteria phage vB_EcoM_VR5 [TAX:1567026]	NC_028881
Enterobacteria phage vB_EcoP_ACG-C91 [TAX:1141139]	NC_019403
Enterobacteria phage vB_EcoS_NBD2 [TAX:1852563]	NC_031050
Enterobacteria phage vB_EcoS_Rogue1 [TAX:1147155]	NC_019718
Enterobacteria phage VT2-Sakai [TAX:97081]	NC_000902

Enterobacteria phage VT2phi_272 [TAX:936054]	NC_028656
Enterobacterial phage mEp213 [TAX:1147156]	NC_019720
Enterobacterial phage mEp234 [TAX:1147157]	NC_019715
Enterobacterial phage mEp390 [TAX:1147158]	NC_019721
Escherichia coli O157 typing phage 1 [TAX:1508671]	KP869100
Escherichia coli O157 typing phage 10 [TAX:1508672]	KP869108
Escherichia coli O157 typing phage 11 [TAX:1508673]	KP869109
Escherichia coli O157 typing phage 12 [TAX:1508674]	KP869110
Escherichia coli O157 typing phage 3 [TAX:1508678]	KP869101
Escherichia coli O157 typing phage 5 [TAX:1508680]	KP869103
Escherichia coli O157 typing phage 6 [TAX:1508681]	KP869104
Escherichia phage 121Q [TAX:1555202]	NC_025447
Escherichia phage 172-1 [TAX:1598146]	NC_028903
Escherichia phage 1720a-02 [TAX:1115653]	KF030445
Escherichia phage 4MG [TAX:1391428]	NC_022968
Escherichia phage 64795_ec1 [TAX:1837842]	NC_031114
Escherichia phage ADB-2 [TAX:1216926]	NC_019725
Escherichia phage APCEc01 [TAX:1655305]	NC_029091
Escherichia phage AR1 [TAX:66711]	NC_027983
Escherichia phage Av-05 [TAX:1527519]	NC_025830
Escherichia phage Bp4 [TAX:1458848]	NC_024142
Escherichia phage bV_EcoS_AHP24 [TAX:1416027]	KF771236
Escherichia phage CAjan [TAX:1610828]	NC_028776
Escherichia phage CICC 80001 [TAX:1527506]	NC_027387
Escherichia phage D108 [TAX:665033]	NC_013594
Escherichia phage e4/1c [TAX:1495286]	NC_024210
Escherichia phage EB49 [TAX:1048207]	NC_023743
Escherichia phage EC1-UPM [TAX:1258572]	KC206276
Escherichia phage EC6 [TAX:1229757]	NC_027369
Escherichia phage ECBP1 [TAX:1604356]	NC_018854
Escherichia phage ECBP2 [TAX:1604355]	NC_018859
Escherichia phage ECBP5 [TAX:1498172]	NC_027330
Escherichia phage ECML-117 [TAX:1204521]	NC_025441
Escherichia phage ECML-134 [TAX:1204522]	NC_025449
Escherichia phage ECML-4 [TAX:1204523]	NC_025446
Escherichia phage EK99P-1 [TAX:1527514]	NC_024783
Escherichia phage Envy [TAX:1883200]	NC_031081
Escherichia phage FFH2 [TAX:1446490]	NC_024134
Escherichia phage FV3 [TAX:1131317]	NC_019517
Escherichia phage Gluttony [TAX:1883202]	NC_031113

Escherichia phage HK578 [TAX:1147142]	NC_019724
Escherichia phage HK639 [TAX:906669]	NC_016158
Escherichia phage HK75 [TAX:906668]	NC_016160
Escherichia phage HX01 [TAX:1237364]	NC_018855
Escherichia phage HY01 [TAX:1434323]	NC_027349
Escherichia phage HY02 [TAX:1527531]	NC_028872
Escherichia phage HY03 [TAX:1654926]	NC_031047
Escherichia phage ime09 [TAX:1054834]	NC_019503
Escherichia phage IME11 [TAX:1239384]	NC_019423
Escherichia phage JES2013 [TAX:1327956]	NC_022323
Escherichia phage JH2 [TAX:1340750]	NC_029023
Escherichia phage JS98 [TAX:293178]	NC_010105
Escherichia phage JSE [TAX:576789]	NC_012740
Escherichia phage K1-ind(2) [TAX:698489]	GU196280
Escherichia phage K1-ind(3) [TAX:698490]	GU196281
Escherichia phage K1ind1 [TAX:698488]	GU196279
Escherichia phage KBNP1711 [TAX:1436889]	NC_023593
Escherichia phage LM33_P1 [TAX:1788294]	NC_031937
Escherichia phage Lw1 [TAX:1307804]	NC_021344
Escherichia phage Min27 [TAX:489779]	NC_010237
Escherichia phage MX01 [TAX:1837930]	NC_031934
Escherichia phage NJ01 [TAX:1237159]	NC_018835
Escherichia phage P13374 [TAX:1150869]	NC_018846
Escherichia phage P483 [TAX:1572753]	NC_028822
Escherichia phage P694 [TAX:1572754]	NC_028863
Escherichia phage PA2 [TAX:1660365]	NC_028449
Escherichia phage PBECO 4 [TAX:1273738]	NC_027364
Escherichia phage PE3-1 [TAX:1498170]	NC_024379
Escherichia phage phAPEC8 [TAX:1229753]	NC_020079
Escherichia phage PhaxI [TAX:926589]	NC_019452
Escherichia phage phi191 [TAX:1458706]	NC_028660
Escherichia phage phiV10 [TAX:343516]	NC_007804
Escherichia phage Pollock [TAX:1540097]	NC_027381
Escherichia phage pro147 [TAX:1649239]	NC_028896
Escherichia phage pro483 [TAX:1649240]	NC_028943
Escherichia phage RB3 [TAX:31533]	NC_025419
Escherichia phage Seurat [TAX:1540098]	NC_027378
Escherichia phage slur01 [TAX:1720493]	NC_028831
Escherichia phage slur02 [TAX:1720494]	NC_028927
Escherichia phage slur05 [TAX:1720498]	NC_028901

Escherichia phage slur14 [TAX:1720504]	NC_028448
Escherichia phage slur16 [TAX:1720495]	NC_028248
Escherichia phage Stx2 II [TAX:194949]	NC_004914
Escherichia phage SUSP1 [TAX:1718606]	NC_028808
Escherichia phage SUSP2 [TAX:1718669]	NC_028935
Escherichia phage TL-2011b [TAX:1124654]	NC_019445
Escherichia phage TL-2011c [TAX:1124655]	NC_019442
Escherichia phage UFV-AREG1 [TAX:1837867]	NC_031030
Escherichia phage rV5 [TAX:399183]	NC_011041
Escherichia phage vB_EcoM-ep3 [TAX:1541883]	NC_025430
Escherichia phage vB_EcoM-UFV13 [TAX:1815590]	NC_031103
Escherichia phage vB_EcoM-VpaE1 [TAX:1555238]	NC_027337
Escherichia phage vB_EcoM_ACG-C40 [TAX:1141141]	NC_019399
Escherichia phage vB_EcoM_Alf5 [TAX:1873990]	NC_031082
Escherichia phage vB_EcoM_AYO145A [TAX:1636202]	NC_028825
Escherichia phage vB_EcoM_ECO1230-10 [TAX:669875]	NC_027995
Escherichia phage vB_EcoM_JS09 [TAX:1430444]	NC_024124
Escherichia phage vB_EcoM_PhAPEC2 [TAX:1391224]	NC_024794
Escherichia phage vB_EcoM_VR20 [TAX:1567027]	NC_028894
Escherichia phage vB_EcoM_VR25 [TAX:1567028]	NC_028925
Escherichia phage vB_EcoM_VR26 [TAX:1567029]	NC_028957
Escherichia phage vB_EcoM_VR7 [TAX:700939]	NC_014792
Escherichia phage vB_EcoP_24B [TAX:866553]	NC_027984
Escherichia phage vB_EcoP_G7C [TAX:1054461]	NC_015933
Escherichia phage vB_EcoP_GA2A [TAX:1755695]	NC_031943
Escherichia phage vB_EcoP_PhAPEC5 [TAX:1395983]	NC_024786
Escherichia phage vB_EcoP_PhAPEC7 [TAX:1391223]	NC_024790
Escherichia phage vB_EcoP_SU10 [TAX:1519788]	NC_027395
Escherichia phage vB_EcoS_AHP42 [TAX:1416028]	NC_024793
Escherichia phage vB_EcoS_AHS24 [TAX:1416030]	NC_024784
Escherichia phage vB_EcoS_AKS96 [TAX:1416031]	NC_024789
Escherichia phage vB_EcoS_FFH1 [TAX:1446489]	NC_024139
Escherichia phage vB_Eco_ACG-M12 [TAX:1141140]	NC_019404
Escherichia phage WG01 [TAX:1837931]	NC_031928
Escherichia phage wV7 [TAX:1054480]	NC_019505
Escherichia phage wV8 [TAX:576791]	NC_012749
Escherichia phage YD-2008.s [TAX:1567004]	NC_027383
Escherichia Stx1 converting phage [TAX:194948]	NC_004913
Escherichia Stx1-converting recombinant phage HUN/2013 [TAX:1506453]	KJ909655
Escherichia virus 186 [TAX:29252]	NC_001317

Escherichia virus AKFV33 [TAX:1112008]	NC_017969
Escherichia virus CBA120 [TAX:1987159]	NC_016570
Escherichia virus DT57C [TAX:1567006]	NC_027356
Escherichia virus EPS7 [TAX:491003]	NC_010583
Escherichia virus HK022 [TAX:10742]	NC_002166
Escherichia virus HK97 [TAX:37554]	NC_002167
Escherichia virus JL1 [TAX:1245890]	NC_019419
Escherichia virus K1-5 [TAX:187764]	NC_008152
Escherichia virus K1E [TAX:344022]	NC_007637
Escherichia virus JK06 [TAX:1920999]	NC_007291
Escherichia virus Lambda [TAX:10710]	NC_001416
Escherichia virus Mu [TAX:10677]	NC_000929
Escherichia virus N15 [TAX:40631]	NC_001901
Escherichia virus N4 [TAX:10752]	NC_008720
Escherichia virus P1 [TAX:10678]	NC_005856
Escherichia virus P2 [TAX:10679]	NC_001895
Escherichia virus phiEco32 [TAX:490103]	NC_010324
Escherichia virus RB16 [TAX:329381]	NC_014467
Escherichia virus RB32 [TAX:45406]	NC_008515
Escherichia virus Rtp [TAX:355246]	NC_007603
Escherichia virus SSL2009a [TAX:624134]	NC_012223
Escherichia virus T1 [TAX:1921008]	NC_005833
Escherichia virus T4 [TAX:10665]	NC_000866
Escherichia virus T5 [TAX:10726]	NC_005859
Escherichia virus TLS [TAX:245685]	NC_009540
Escherichia virus Wphi [TAX:103216]	NC_005056
Salmonella phage SFP10 [TAX:1080800]	NC_016073
Salmonella virus HK620 [TAX:155148]	NC_002730
Salmonella virus PRD1 [TAX:10658]	NC_001421
Salmonella virus SPC35 [TAX:977927]	NC_015269
Stx2 converting phage I [TAX:180816]	AP004402
Stx2-converting phage 1717 [TAX:563769]	NC_011357
Stx2-converting phage 86 [TAX:379329]	NC_008464
Escherichia phage phiKT	NC_019520
Escherichia phage slur07	NC_028780
Escherichia phage K1G	NC_027993
Escherichia phage K1H	NC_027994
Enterobacteria phage K30	NC_015719
Enterobacteria phage QL01	NC_028847
Escherichia phage slur09 genome assembly slu09	NC_028840

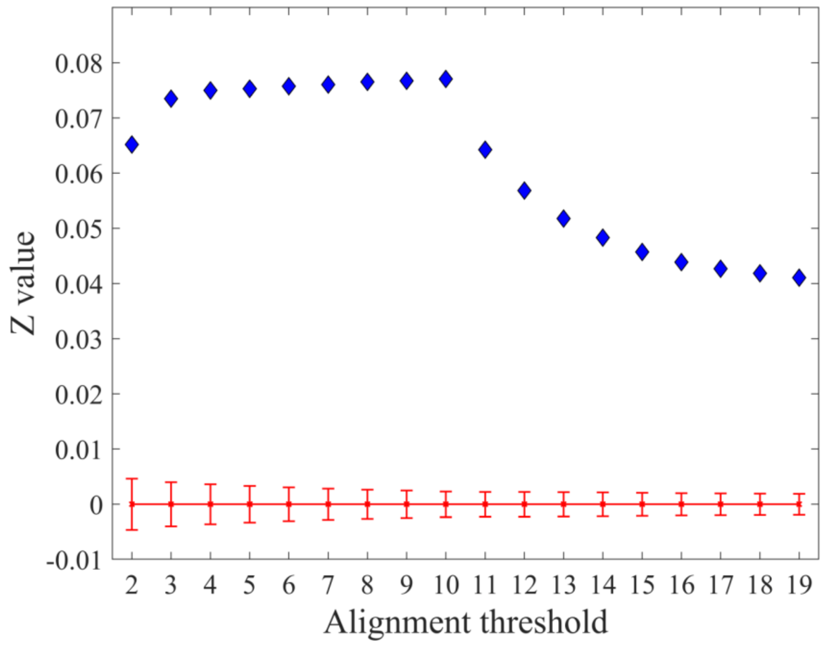

Figure S1. Z value expressed as a function of the number of alignments; Z value corresponds to the difference between the mean SAS value of the *E. coli* hits vs. randomized *E. coli* hits divided by the mean SAS value of the randomized *E. coli* hits (shown on the Figure as blue diamond); Red vertical bars correspond to the confidence bound estimates of the analogously defined Z value for the randomized background. Note that the Z values for *E. coli* hits are significantly higher compared to the randomized background, over the entire threshold range.

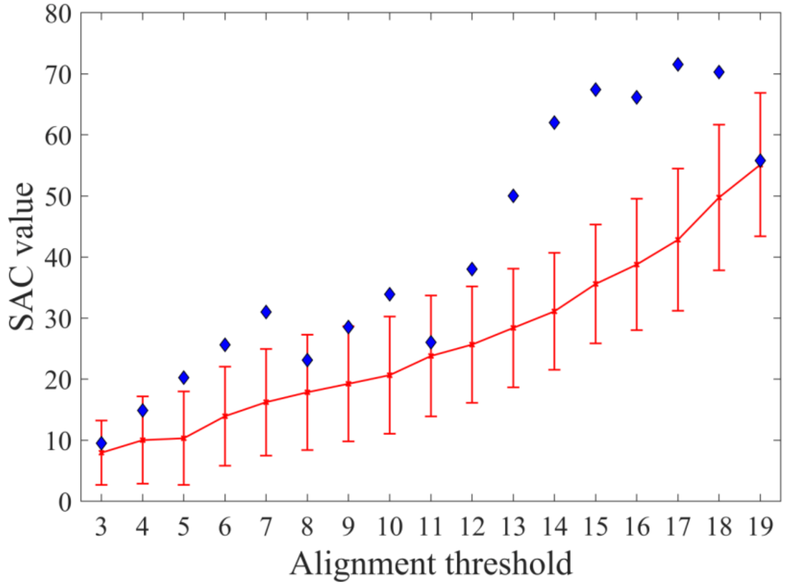

Figure S2. SAC value expressed as a function of the number of alignments; SAC value (shown as a blue diamond) here corresponds to the difference between the SAC value of coding regions and weighted SAC value of intergenic regions. The weighting coefficient is obtained by dividing the full length of coding regions by the full length of intergenic regions of the *E. coli* genome (w = 5.6235); Red vertical bars correspond to the confidence bound estimates of the analogously defined SAC value for the randomized background.
